# Voltammetric Determination of the Herbicide *Linuron* Using a Tricresyl Phosphate-Based Carbon Paste Electrode

**DOI:** 10.3390/s120100148

**Published:** 2011-12-22

**Authors:** Jelena Đorđević, Zsigmond Papp, Valéria Guzsvány, Ivan Švancara, Tatjana Trtić-Petrović, Milovan Purenović, Karel Vytřas

**Affiliations:** 1 Laboratory of Physics, Vinča Institute of Nuclear Sciences, P.O. Box 522, 11001 Belgrade, Serbia; E-Mails: jdjordjevic@vin.bg.ac.rs (J.Đ.); ttrtic@vinca.rs (T.T.-P.); 2 Department of Chemistry, Biochemistry and Environmental Protection, Faculty of Sciences, University of Novi Sad, Trg D. Obradovića 3, 21000 Novi Sad, Serbia; E-Mail: zsigmond.papp@dh.uns.ac.rs; 3 Department of Analytical Chemistry, University of Pardubice, Studentská 573, 53210 Pardubice, Czech Republic; E-Mails: Ivan.Svancara@upce.cz (I.Š.); karel.vytras@upce.cz (K.V.); 4 Department of Chemistry, Faculty of Sciences and Mathematics, University of Niš, P.O. Box 224, 18000 Niš, Serbia; E-Mail: puren@pmf.ni.ac.rs

**Keywords:** *Linuron*, herbicide, carbon paste electrode, tricresyl phosphate, differential pulse voltammetry

## Abstract

This paper summarises the results of voltammetric studies on the herbicide 3-(3,4-dichlorophenyl)-1-methoxy-1-methylurea (*Linuron*), using a carbon paste electrode containing tricresyl phosphate (TCP-CPE) as liquid binder. The principal experimental conditions, such as the pH effect, investigated in Britton-Robinson buffer solutions (pH 2.0–7.0), the peak characteristics for the analyte of interest, or instrumental parameters for the differential pulse voltammetric mode were optimized for the method. As found out, the best electroanalytical performance of the TCP-CPE was achieved at pH 2.0, whereby the oxidation peak of *Linuron* appeared at *ca.* +1.3 V *vs.* SCE. The analytical procedure developed offers good linearity in the concentration range of 1.25–44.20 μg mL^−1^ (1.77 × 10^−4^–5.05 × 10^−6^ mol L^−1^), showing—for the first time—the applicability of the TCP-CPE for anodic oxidations in direct voltammetry (without accumulation). The method was then verified by determining *Linuron* in a spiked river water sample and a commercial formulation and the results obtained agreed well with those obtained by the reference HPLC/UV determination.

## 1. Introduction

**Figure 1.**
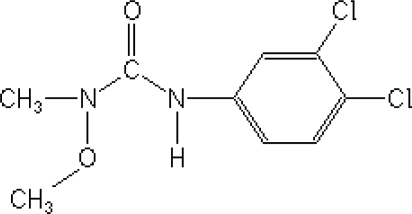
Chemical formula of *Linuron.*

Pesticides, where used correctly, can save up to 40% of crop losses; however, when pesticides are mal-, mis- or over-used, the environmental and public health consequences can be quite severe [1]. Phenylurea herbicides are among the most widely used herbicides in non-crop areas, as well as in tree crops [2]. *Linuron* [3-(3,4-dichlorophenyl)-1-methoxy-1-methylurea, Figure 1] is a herbicide from the substituted phenylurea family that is used widely for selective control of broadleaf weeds and grasses in fruit or field crops, cereals, and shelter belts [3]. The half-life of *Linuron* in soil is *ca*. from 40 to 70 days [2], and the ground water may retain milligram concentrations for several days and weeks [4], depending on the water temperature and pH.

A wide spectrum of analytical methods has been applied to the analysis of pesticides; most of them being based on chromatographic techniques [5,6]. Phenylurea herbicides cannot be analyzed directly by gas chromatography due to their polar and thermolabile nature [7,8], therefore, high performance liquid chromatography (HPLC) is preferably used for phenylurea analysis, often with UV or diode array detection [9–11]. Other detection systems, such as photolysis cells in series with a fluorescence detector [12], post-column derivatization system [13], electrochemical detectors [14], and mass spectrometry [15,16] have been coupled with HPLC to analyze these compounds. However, HPLC is both instrumentally and financially demanding and the respective analysis may be time-consuming.

Compared to these techniques modern voltammetric methods—in combination with a large variety of electrodes and other detection systems [17,18]—are usually simpler, inexpensive, rapid, as well as sufficiently sensitive and selective to be employed for a large-scale monitoring of electrochemically active environmental pollutants [17]. Among the electrodes of choice, the so-called *carbon paste electrodes* (CPEs [19–22]) offer a number of advantageous features; namely, simple preparation (often in labs in a wide palette of various configurations, including very special mixtures), minimal cost, favorable signal-to-noise characteristics (in both faradic and non-faradic measurements), unique surface characteristics, and mainly, almost unlimited possibilities for chemical and biological modification [21,23–25]. When one considers also their enviromentally friendly character, wide adaptability to the latest trends, or a full compatibility with miniaturised detection systems and modern technologies, it is not so surprising that these electrodes are so frequently employed in analysis of pesticides and related substances (see [22] and refs. therein).

This is also the case of hitherto-known voltammetric studies dealing with *Linuron*, where CPEs have represented a fully comparable alternative to other carbon-based electrodes [26–29], including some micro- and ultramicro-configurations [28,29].

One of “special” carbon pastes mentioned above as possible alternative is a mixture of graphite powder and liquid tricresyl phoshate (TCP), introduced into electrochemistry with CPEs in the early 1990s [30,31]. The configuration of the TCP-CPE was inspired by research work by Kalcher, who had employed similar substances as liquid ion-exchangers properties intentionally selected for modification of carbon paste [32–34], as well as by previous use of TCP as the plasticizer for liquid membranes of some ion-selective electrodes [35]. The resultant TCP-CPE is a typical representative of a CPE with chemically active binder, where the molecules of organophoshate can readily be protonated [36]:

(1a) $${\left(C_{7}H_{7}O\right)}_{3}P=O+H^{+}\to {\left[{\left(C_{7}H_{7}O\right)}_{3}P=\text{OH}\right]}^{+}$$

and, in such a form, used in some ion-exchange/ion-pairing processes, e.g.,:

(1b) $${\left[{\left(C_{7}H_{7}O\right)}_{3}P=\text{OH}\right]}^{+}+Y^{-}\to \{{\left[{\left(C_{7}H_{7}O\right)}_{3}P=\text{OH}\right]}^{+};\;{\left[Y\right]}^{-}\}$$

giving a relatively stable and electroactive ion-associate. From then onwards, the TCP-CPE has been successfully employed in numerous electroanalytical methods, utilising the principles from the above Equation (1a) and (1b) and in combination with either voltammetric detection or potentiometric indication. More specifically, the TCP-CPE was the electrode of choice for the determination of gold in the form of [AuCl_4_]^−^ (see [31,37]), silver ions down to the picomolar level [38], bismuth as [BiI_4_]^−^ (see again [31]), thallium via [TlCl_4_]^−^ (see [39]), or numerous anions like BF_4_^−^, ClO_4_^−^, HAsO_4_^2–^, [P(Mo_3_O_10_)_4_]^3−^ (see [40] and refs. therein), and mainly for iodide (as I^−^ or I_3_^−^, respectively; consult e.g., [36] or [41]). Moreover, the TCP-CPE could also be used for some applied studies, which is the case of the microscopy study of the surface morfology of different carbon pastes [42], or the evaluation of stability constants for aurate(III) halides of the [AuX_4_]^−^ type (where “X” is F, Cl, Br, SCN, and CN; see [43]). Regarding organic substances and biologically active compounds, the tricresyl-phosphate based CPEs had so far been tested in some preliminary assays with polyaromatic nitrocompounds, PANs [44] and an (unsuccessful) accumulation study with 6-benzylaminopurine (a plant hormone; see [45]). The group of organic compounds determinable with TCP-CPE can finally be completed with recent investigations on selected neonicotinoids [46–50] and the phenylurea herbicide *Linuron*, reported for the first time in this article.

Thus, the main goal of this work was the basic voltammetric characterization of *Linuron* and subsequently, the elaboration of a simple and effective method for its determination at the TCP-CPE, when using a Britton-Robinson buffer as the supporting electrolyte of choice. The resultant differential pulse voltammetric method could then be successfully applied to determine the herbicide in model solutions, a spiked river water sample and in a specimen of a commercial formulation. All the important observations and results are given in the following sections.

## 2. Experimental Procedures

### 2.1. Chemicals and Solutions

*Linuron* (purity 95% w/w) was obtained from Galenika-Fitofarmacija A.D. (Serbia). The concentration of the *Linuron* stock solution—prepared by dissolving this herbicide in methanol (Sigma-Aldrich)—was 2,500 μg mL^−1^, and it was further diluted as required. As supporting electrolytes, BR buffer solutions of different pHs (between 2.0 and 7.0) were prepared by mixing solutions of 0.04 mol L^−1^ H_3_PO_4_ (Merck), 0.04 mol L^−1^ H_3_BO_3_ (Merck), and 0.04 mol L^−1^ CH_3_COOH (Merck) and adjusting pH by adding suitable amounts of 0.2 mol L^−1^ NaOH (Merck). All other reagents were of analytical reagent grade and solutions were prepared in doubly distilled water. For the preparation of the mobile phase in HPLC experiments, methanol (Sigma-Aldrich, Germany) and doubly distilled water were used. The river water sample was collected from the Tisa River (Senta, Serbia) and stored in the dark at *ca.* 4 °C for one week before analysis.

### 2.2. Apparatus

Voltammetric experiments were performed on an Autolab (PGSTAT12, Ecochemie, The Netherlands) electrochemical analyzer operated via the GPES 4.9 software from the same manufacturer. The cell stand included a three-electrode system with a TCP-CPE as working electrode, a saturated calomel electrode (SCE, Amel, Italy) as the reference, and a platinum auxiliary electrode (Amel). All potentials are quoted *vs*. SCE reference electrode. A PC–controlled magnetic stirrer was employed rotating at approx. 300 rpm. All electrochemical experiments were carried out in a one-compartment 20 mL^−1^ voltammetric cell at room temperature at *ca.* 23 ± 1 °C. The reference HPLC measurements were performed using a Dionex-0650 liquid chromatograph (Dionex, USA), Agilent Eclipse XDB-C18 (4.6 mm × 250 mm, 3.5 μm) column, and a UV-detector.

### 2.3. Procedures

*Preparation of CPE.* Carbon paste was made by thorough hand-mixing CR 5 graphite powder (Maziva Týn, Czech Republic, 0.25 g) with 0.1 mL tricresyl phosphate (mixture of isomers, Sigma-Aldrich Chemie GmbH, Switzerland) as the pasting liquid. The detailed procedure of the electrode preparation was described earlier [46]. The paste was packed into a piston-driven Teflon^®^ holder [51]. The electrode surface of CPEs (2 mm in diameter) was renewed mechanically by smoothing some paste off with a wet piece of filter paper before starting a new set of experiments.

*Voltammetry on TCP-CPE.* Solutions were measured in 10.00 mL of Britton-Robinson (BR) buffer supporting electrolyte to which different concentrations of *Linuron* was added. The solutions were deaerated by passing a nitrogen stream through them for 5 min. The voltammograms were recorded at room temperature. Before each measurement, the buffer-immersed working electrode was electrochemically activated by potential cycling (with 50 cycles) in the range from −0.1 to +1.6 V using a scan rate, *v* = 0.25 V s^−1^. Before measuring *Linuron*, the blank signal was recorded in the supporting electrolyte. The scan rate in the cyclic voltammetric (CV) investigation of *Linuron* was 25 mV s^−1^. The DPV measurement parameters were as follows: start potential −0.1 V, end potential +1.6 V, pulse amplitude 50 mV, pulse width 50 ms, pulse time of 0.05 s, and scan rate 25 mV s^−1^. The model solutions and also the river water sample were measured without filtering.

*Chromatography.* For the HPLC/UV analysis, all aliquots were filtered through Restek 0.22 μm syringe filters. The mobile phase was a mixture of methanol and doubly distilled water in ratio 7:3, v/v. The separation was performed in the isocratic regime and the flow rate was 0.8 mL min^−1^. *Linuron* was detected at a wavelength of 220 nm with the retention time of 12 min.

*Real Sample Preparation.* Because HPLC analysis did not confirm the presence of detectable amounts of the target compound, the river water sample was spiked with the standard solution of *Linuron* to achieve a concentration of 14.92 μg mL^−1^. The sample was kept in the dark at *ca*. 4 °C for 1 h before analysis without any sample pretreatment. Before DPV measurements the aliquots of the spiked river water sample were diluted with BR buffer pH 2.0 in ratio 1:1 v/v. The commercial formulation of *Linuron*, Galolin mono, was dissolved in methanol and further diluted with BR buffer pH 2.0 to the required concentration. Filtering was performed only before HPLC measurements.

## 3. Results and Discussion

### 3.1. Voltammetric Investigation of Linuron at TCP-CPE

**Figure 2.**
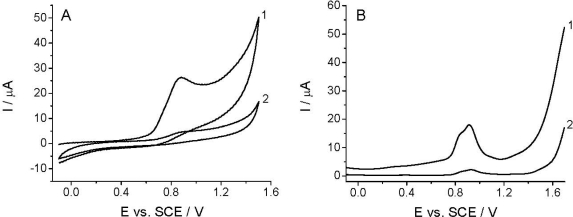
The effect of potential cycling on the TCP-CPE baseline in CV (**A**) and DPV (**B**) experiments at pH 2.0: baseline without cycling (1) and after 10 cycles (2).

The voltammetric detection, as well as the determination of *Linuron* is based on its oxidation at the working electrode [26–29]. Among others, the quality of the analytical signal depens on the nature of working electrode, electrode pretreatment, supporing electrolyte, and the compound investigated. Figure 2 shows that the CV and DPV curves (curves 1) obtained in the BR buffer solution at pH 2.0 recorded with TCP-CPE working electrode contained one irreversible oxidation peak with Ep at around +0.9 V. The oxidation peak is wide and covers the potential range between +0.65 and +1.10 V, which makes difficult to measure within this potential range. This undesirable signal is also present in some another electrolytes (phosphate, sulfate, acetate), and it is assumed to be a result of the oxidation of the working electrode surface. Additionally, the residual current is too high; especially, from +0.7, narrowing the accessible potential window of the working electrode. As well known, electrochemical pretreatment (also termed electrochemical activation) of the working electrode surface is a widely used procedure to enhance sensitivity and selectivity in voltammetric analyses of organic compounds [52]. Both anodic (or cathodic) polarization performed at extreme potentials and anodic-cathodic cycling, are commonly employed procedures to pre-treat the surface of carbon electrodes, including CPEs [53,54], for which one can also utilize a special “surface erosion” with surfactants [55]. In order to obtain reproducible response, a lower residual current and wider potential window in the anodic range the TCP-CPE working electrode was subjected to electrochemical activation by potential cycling in the range from −0.1 to +1.60 V (10 cycles, *v* = 0.25 V s^−1^) in the blank prior to measurements. This pretreatment led to a significant improvement in the baseline characteristics in the case of both CV and DPV measurements (Figure 2, curves 2). Increasing the number of potential cycles (curves) lead to a further widening of the potential window, decreases of the mentioned oxidation peak an additional signal stabilization. During the electrochemical treatment the TCP-CPE did not lose its sensitivity.

**Figure 3.**
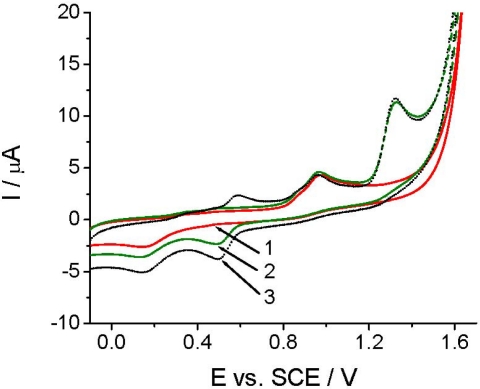
Cyclic voltammetric investigation of 36.95 μg mL^−1^ *Linuron* using a TCP-CPE at pH 2.0: baseline (1), the first cycle (2), and the second cycle (3).

Before applying TCP-CPE for quantitative determinations, it was also necessary to perform characterization of the target compound. As demonstrated at pH 2.0 (Figure 3), *Linuron* exhibited a rather complex electrochemical behavior. On the first cyclic voltammogram (curve 2), in addition to the peaks at +0.14 and +0.95 V, which are also present in the blank (curve 1), a well defined *Linuron* oxidation peak appears at +1.32 V. The oxidation of *Linuron* resulted in the appearance of a new reduction signal at around +0.5 V in the second potential half-cycle (Figure 3, curve 2). In the second cycle (after solution stirring), a counterpart of this signal appeared in the positive scan at *ca.* +0.6 V (Figure 3, curve 3). The appearance of these new signals corresponded possibly to the degradation products of *Linuro*n [29]. The appearance of the new signals did not affected the intensity of the analytical signal, neither in CV nor in DPV mode, which allowed us to choose a simpler direct voltammetric determination also for achieving a good reproducibility.

**Figure 4.**
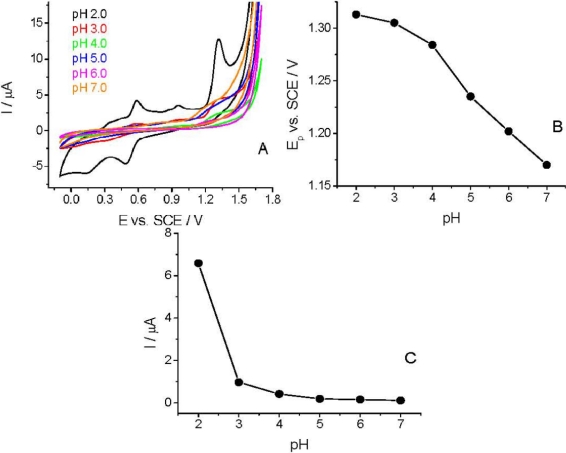
CV signals (second cycles) of 36.95 μg mL^−1^ *Linuron* at different pHs **(A)**, and the variation of the peak potential **(B)** and peak current **(C)** of *Linuron* with pH at TCP-CPE in CV experiments.

The CV curves were recorded in the pH range of 2.0–7.0 to study the effect of pH on the voltammetric behavior of *Linuron*. The sharpest and most favorably developed oxidation peak for *Linuron* was obtained in strongly acidic solution (pH 2.0). As clearly visible (Figure 4), both peak potential and peak current intensity depend on the pH of the supporting electrolyte. At pH > 3.0 the peak potential (Ep) is more pH-dependent than at lower pHs. This pH-dependency indicates the involvement of protons in the electrode reaction and that the proton-transfer reaction precedes the electrode process proper. Similarly to the earlier published experimental data [28,29], the Ep–pH plot [Figure 4(B)] for the main oxidation peak shows two linear parts with a break at approximately pH 4.0; the slope of the first part being 14.5 mV pH^−1^ and of the second one 37.5 mV pH^−1^.

### 3.2. Direct Anodic DPV Determination of Linuron at TCP-CPE

**Figure 5.**
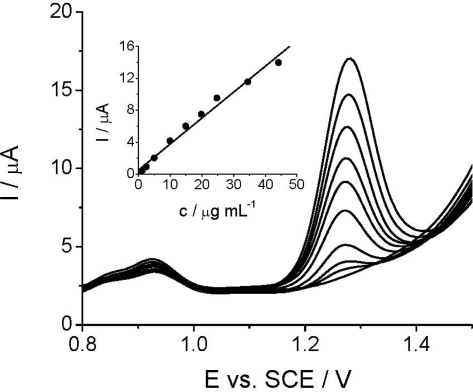
Differential pulse voltammograms recorded at the TCP-CPE for different concentrations of *Linuron* in Britton-Robinson buffer (pH 2.0). The corresponding calibration plot is shown in the inset.

The quantitative DPV determination of *Linuron* at TCP-CPE is based on the linear relationship between the peak current intensity at +1.3 V and *Linuron* concentration. As can be seen, *Linuron* could be determined by DPV in the concentration range of 1.25–44.20 μg mL^−1^ (1.77 × 10^−4^–5.05 × 10^−6^ mol L^−1^) (Figure 5), while the relative standard deviation (RSD) did not exceed 2.7%. The analytical parameters of the developed DPV method and the comparative HPLC/UV measurements are shown in the Table 1.

**Figure 6.**
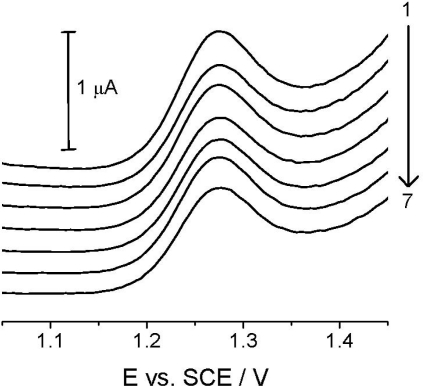
Reproducibility of the analytical signal for 2.5 μg mL^−1^ of *Linuron* at TCP-CPE in *ca.* 30 min time interval.

Reproducibility studies at TCP-CPE were also performed, first of all to check the signal stability and possible changes in its shape because of potentially approaching absorption/adsorption processes. Figure 6, in which records are presented for seven repeated measurements of the 4.99 μg mL^−1^ (2.00 × 10^−5^ mol L^−1^) *Linuron* solution, showing good reproducibility of the analytical signal in the time interval of approx. 30 minutes, with no significant changes in the electrode properties and the analyte signal itself during the experimentation.

**Figure 7.**
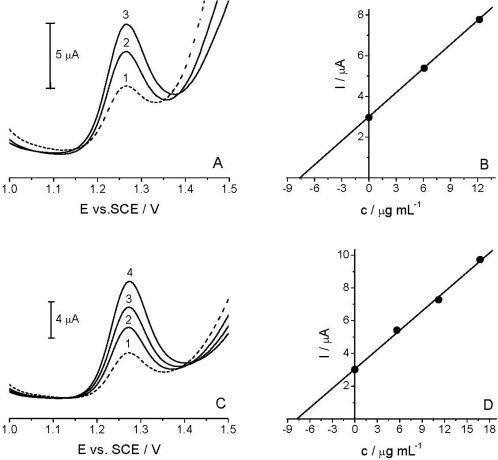
Determination of *Linuron* in real samples. Spiked river water sample **(A)**: with the corresponding standard addition plot **(B)**: spiked sample (1) and successive standard additions (2, 3), and commercial formulation Galolin Mono **(C)**: with the corresponding standard addition plot **(D)**: sample (1) and successive standard additions (2–4).

The applicability of the voltammetric procedure was tested by the determination of the *Linuron* in the spiked river water sample [Figure 7(A)] and commercial formulation Galolin mono [Figure 7(C)], using DPV with TCP-CPE.

As HPLC/UV measurements did not confirm the presence of *Linuron* in the analyzed river water sample, in subsequent experiments a spiked sample (14.92 μg mL^−1^) was used. As described in Section 2.3, the sample was diluted with BR pH 2.0 at a 1:1 v/v ratio. The standard addition method was applied for the determination of the target compound [Figure 7(B)]. Like in the river water analysis, the content of the active compound in the analyzed commercial formulation was determined by the standard addition method [Figure 7(D)]. Good correlation between the quantity determined and the amount added/declared, as well as fairly low RSD, reflected both the high accuracy and precision of the proposed method (see Table 2). Finally, the results obtained by DPV method agreed well with the comparative HPLC/UV analysis (Table 2). Thus, it can be stated that further experiments are needed to further extend the applicability of the TCP-CPE for anodic oxidations, as well as to achieve yet better detection parameters for particularly low concentrations of the target compound in some environmental samples.

## 4. Conclusions

In this article, the tricresyl phosphate-based carbon paste electrode, TCP-CPE, has been shown to be the electrode of choice for the determination of an organic pollutant—the herbicide *Linuron*. As demonstrated and discussed in the previous sections, the respective quantitative measurements can be characterized by all principal features needed for the use of the method developed in environmental analysis. Moreover, the TCP-CPE—as a typical representative of special carbon paste electrodes with the chemically active binder [21]—has been employed for the first time in direct voltammetry (*i.e*., without an accumulation step); in this case, for anodic oxidation at a very high positive potential, when using a sensitive differential pulse potential ramp.

Thus, in contrast to many previous procedures employing the TCP-CPE in electrochemical stripping analysis (ESA) with pre-concentration step (see [31,36–39,43–45]), there is yet another interesting aspect of this method that is associated with direct measurements and that has not been described above. It is a lowered risk of unwanted saturation of the TCP-CPE surface layer which is among the less attractive features of this special CPE and which has been observed repeatedly (see e.g., [36,38,41]) as a spontaneous extraction of the analyte into the deep carbon paste bulk, requiring afterwards a very thorough removal of so “infected” paste (up to several mms of paste portion extruded from the body). On the other hand, the use of some of ESA techniques with an incorporated accumulation step would surely lead to an improvement in detection capabilities of the TCP-CPE towards presented herein for direct measurements. Then, there will be a strong motivation for continuing investigations with this electrode in practical electroanalysis—to find and define a compromise in using TCP-CPE within an ESA procedure. It seems that it could be accomplished by means of pre-concentration for a very short period (e.g., 5–10 s), for which the specific saturation inside the TCP-based carbon pastes would not be so pronounced.

## Figures and Tables

**Table 1.** Analytical parameters of the DPV and HPLC/UV determination of *Linuron*.

**Parameter**	**Method (employing)**	
	**DPV**	**HPLC/UV**
Concentration interval [μg mL^−1^]	1.25–44.20	0.125–10.00
Intercept	0.5126 μA	2.5279 mAU min
Slope	0.3249 μA mL μg^−1^	97.1291 mAU min mL μg^−1^
Correlation coefficient, *r*	0.992	0.999
Limit of detection, LOD [μg mL^−1^]	0.38	0.0375
Limit of quantitation, LOQ [μg mL^−1^]	1.25	0.125
RSD [%] (*n* = 7)	2.7	1.1

**Table 2.** Assay of *Linuron* in real samples (*n* = 3).

**Analyte**	**Technique (For Determination)**			
	**DPV**		**HPLC/UV**	
	**Found**	**RSD/%**	**Found**	**RSD/%**
River water^a^	15.30 μg mL^−1^	3.2	14.97 μg mL^−1^	1.7
Galolin mono^b^	486.6 g L^−1^	5.7	480.1 g L^−1^	4.2

a The added value was 14.92 μg mL^−1^;

b Nominal value 500 ± 25 g L^−1^.
